# Acute bacterial endophthalmitis with focal vitritis at a nasal chandelier illumination port after scleral buckling

**DOI:** 10.1016/j.idcr.2026.e02778

**Published:** 2026-09-23

**Authors:** Yuki Mori, Tomohiko Torikai, Atsushi Shiraishi

**Affiliations:** Department of Ophthalmology, Ehime University Graduate School of Medicine, 454 Shizukawa, Toon, Ehime, 791-0295, Japan

**Keywords:** Scleral buckling, Chandelier illumination, Bacterial endophthalmitis, Surgical complications

## Abstract

**Background:**

Chandelier-assisted scleral buckling (CSB) has been reported to offer enhanced fundus visualization and reduced operative time during retinal detachment repair compared with conventional methods. However, the required intraocular penetration creates a potential route for bacterial entry into the eye.

**Case presentation:**

A 31-year-old man with high myopia presented with a rhegmatogenous retinal detachment involving the macula. He underwent urgent CSB using a 27-gauge chandelier positioned nasally at 5 o′clock. On postoperative day (POD) 10, a localized vitreous opacity appeared precisely at the insertion site. By POD 11, the opacity extended into the inferior vitreous cavity, consistent with acute postoperative endophthalmitis. Despite empirical intravitreal injections of vancomycin and ceftazidime with intensive topical moxifloxacin, the patient developed photophobia, ocular pain, and worsening vitritis on POD 12. An urgent lens-sparing pars plana vitrectomy was performed, and vitreous fluid cultures identified *Staphylococcus epidermidis*. Postoperatively, the inflammation subsided, and at 3 months, full retinal attachment was observed with a visual acuity of 20/16.

**Conclusion:**

Whereas previously reported cases of CSB-related endophthalmitis typically presented with widespread intraocular inflammation obscuring the primary infectious focus, this case captures the early focal progression of vitritis in a chronological sequence. The localization at the nasal chandelier port provides visual clues to a possible entry route. The appearance of a localized vitreous opacity corresponding to a chandelier insertion site—even if subtle—should raise suspicion of acute endophthalmitis and prompt timely evaluation and treatment. The route and timing of bacterial entry cannot be established from this case alone.

A 31-year-old Japanese man with high myopia presented with a macula-involving rhegmatogenous retinal detachment in the right eye (Fig. 1A). Visual acuity was 20/20. Scleral buckling was selected to preserve the lens; chandelier-assisted scleral buckling (CSB) was considered suitable because the single retinal break allowed the chandelier to remain in a fixed position. During urgent CSB, a 27-gauge valved trocar cannula and a single-fiber chandelier endoilluminator (Vivid; Synergetics, O′Fallon, MO, USA) were positioned nasally at 5 o′clock (Fig. 1B). Because the surgery was urgent, no preoperative topical antibiotics were administered. The periocular skin was disinfected with undiluted povidone-iodine and the ocular surface irrigated with six-fold diluted polyvinyl alcohol–iodine; postoperative topical gatifloxacin was administered four times daily. On postoperative day (POD) 5, additional subretinal fluid drainage and buckle repositioning were performed. No intraocular infusion was used during either buckling procedure.

**Fig. 1:**
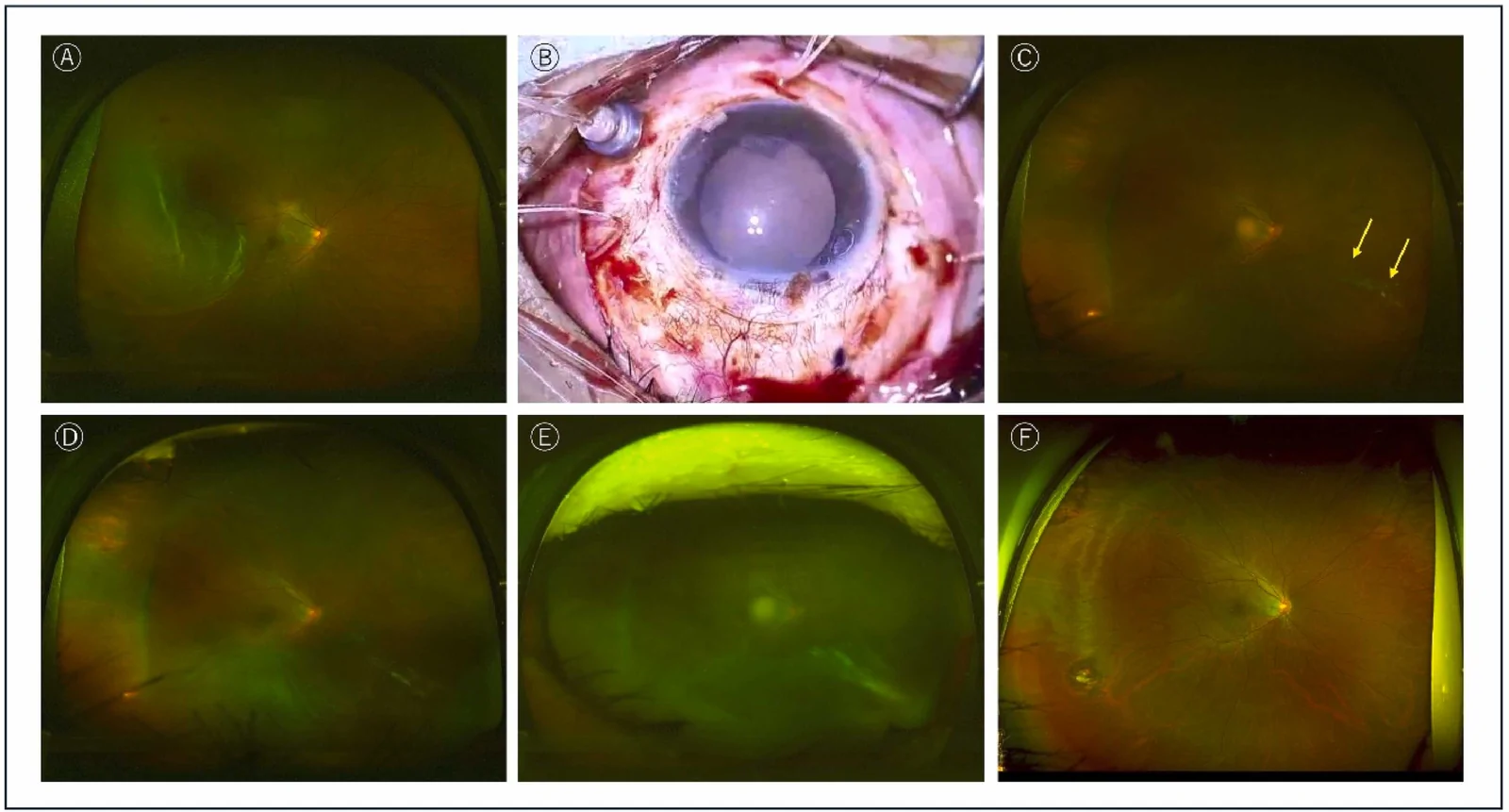
(A) Fundus photograph showing a macula-involving rhegmatogenous retinal detachment in the right eye. (B) Intraoperative view showing the 27-gauge chandelier endoilluminator positioned nasally at the 5 o'clock position during chandelier-assisted scleral buckling. (C) On postoperative day (POD) 10, a localized vitreous opacity appeared precisely at the inferonasal chandelier insertion site (yellow arrows). (D) By POD 11, the opacity had extended into the inferior vitreous cavity. (E) On POD 12, vitritis had further worsened. (F) At 3 months after vitrectomy, the retina was fully attached with a visual acuity of 20/16.

On POD 10, a localized vitreous opacity appeared precisely at the inferonasal chandelier insertion site (Fig. 1C, yellow arrows). By POD 11, the opacity extended to the inferior vitreous cavity (Fig. 1D), consistent with acute postoperative endophthalmitis. Despite empirical intravitreal vancomycin (1 mg) and ceftazidime (2 mg) with hourly topical moxifloxacin, the patient developed photophobia, ocular pain, and worsening vitritis on POD 12 (Fig. 1E), and urgent lens-sparing pars plana vitrectomy was performed. Vancomycin (20 µg/mL) and ceftazidime (40 µg/mL) were administered through the infusion without an additional intravitreal bolus. Vitreous cultures yielded *Staphylococcus epidermidis* susceptible to both agents and moxifloxacin; methicillin susceptibility was not tested. The inflammation subsided, and at 3 months after vitrectomy the retina was fully attached, with visual acuity of 20/16 (Fig. 1F).

Endophthalmitis was reported after 2 of 10,000 conventional scleral buckling procedures [1], whereas none was reported in a meta-analysis of 1133 CSB eyes [2], precluding direct comparison of incidence. In previously reported CSB-related cases, including that of Choudhary et al. [3], diffuse vitritis obscured the primary focus. Transconjunctival trocar insertion may introduce conjunctival flora into the vitreous [4]. The focal opacity and *S. epidermidis* isolate are compatible with this route, but the route and timing of entry remain uncertain. Given the reported susceptibility, progression after intravitreal antibiotics cannot be attributed to resistance. Whether early intervention contributed to the favorable outcome cannot be determined from a single case. A localized vitreous opacity at a chandelier insertion site should raise suspicion of acute endophthalmitis and prompt timely treatment.

## CRediT authorship contribution statement

**Yuki Mori:** Writing – original draft, Investigation, Data curation. **Tomohiko Torikai:** Writing – review & editing, Methodology, Investigation, Data curation, Conceptualization. **Atsushi Shiraishi:** Supervision, Methodology.

## Ethics declaration

Written informed consent to take part in the study and to publish the article has been obtained from all participants or their legal representatives. The privacy rights of participants have been observed.

This study was performed in compliance with relevant laws, regulatory frameworks and guidelines where the research took place. Ethics committee approval was not required under relevant laws and institutional guidelines. This report describes the clinical course of a single patient. Under the Ethical Guidelines for Medical and Biological Research Involving Human Subjects in Japan, a single case report is not regarded as research requiring review by an institutional ethics committee. The report adhered to the tenets of the Declaration of Helsinki, and written informed consent for publication of the case and images was obtained from the patient.

This research follows the CARE guidelines and the CARE checklist.

## Patient consent

Written informed consent was obtained from the patient for publication of this case report and accompanying images. A copy of the written consent is available for review by the Editor-in-Chief of this journal on request.

## Declaration of Generative AI and AI-assisted technologies in the writing process

During the preparation of this work, the authors used Gemini (version 1.5 Pro; Google), ChatGPT (OpenAI), and Claude (Opus 5; Anthropic) to improve readability and language and to check the internal consistency of the manuscript. After using these tools, the authors reviewed and edited the content as needed and take full responsibility for the content of the published article.

## Funding

This work received no specific grant from any funding agency in the public, commercial, or not-for-profit sectors. No study sponsor was involved in the collection, analysis, or interpretation of data, in the writing of the manuscript, or in the decision to submit the manuscript for publication.

## Declaration of interests

We declare no competing interests.
